# Supplementary material for: Phosphoproteomics identifies microglial Siglec‐F inflammatory response during neurodegeneration
Source: Mol Syst Biol. 2020 Dec 8;16(12):e9819. doi: 10.15252/msb.20209819 (PMC7722784; doi:10.15252/msb.20209819)
Supplement: Supplementary file 2 — Expanded View Figures PDF [file MSB-16-e9819-s001.pdf]

Expanded View Figures

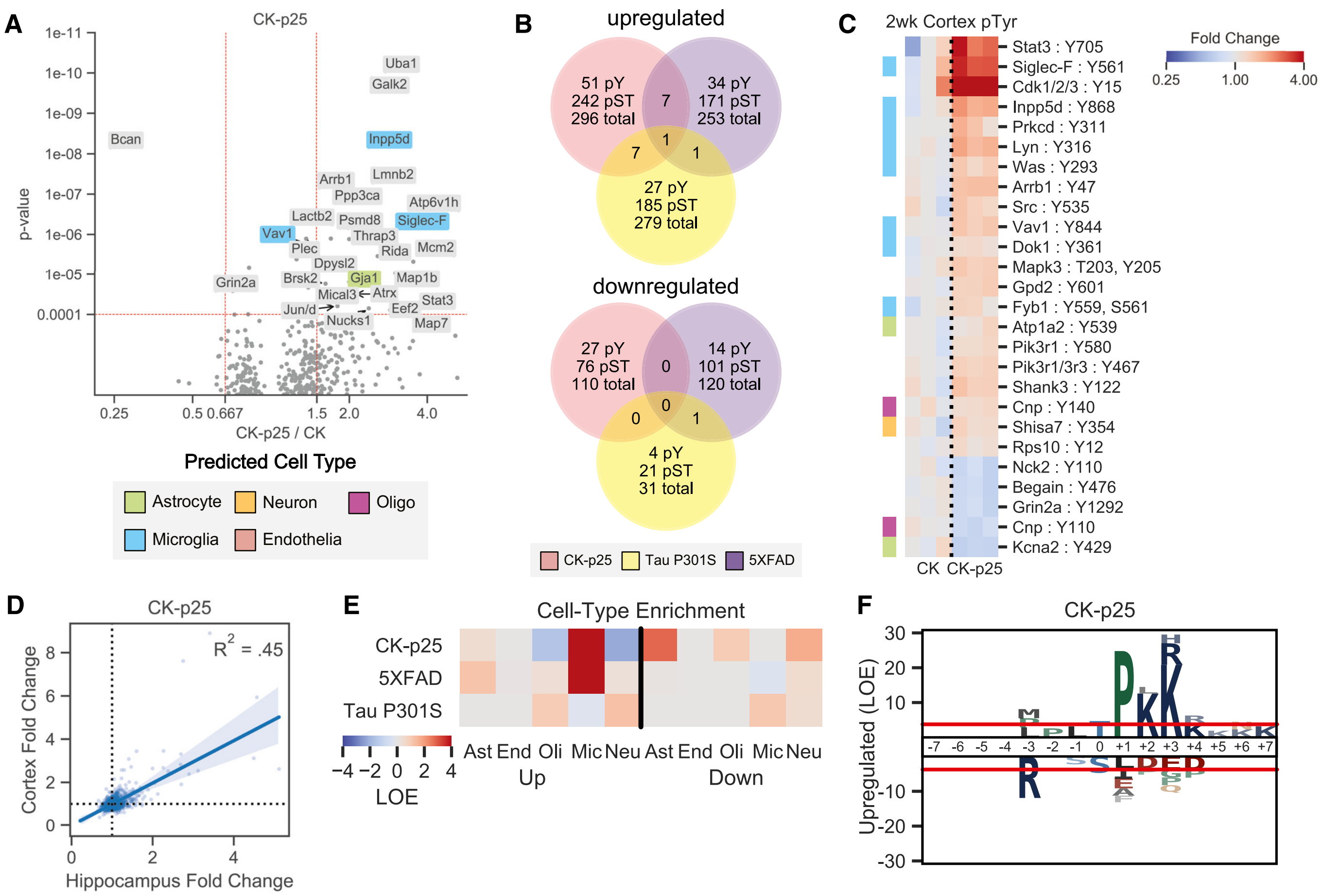

Figure EV1. CK-p25 phosphoproteome accessory analyses.

A Volcano plots showing changed phosphopeptides in CK-p25 mice. All tissues and time points are considered together for fold change and *P*-value calculations. Protein names are shown for changed peptides. Labels are only shown for peptides with maximum directional change from each protein. Labels are colored by predicted cell-type specific protein. Green = Astrocyte, Orange = Neuron, Blue = Microglia, Purple = Oligodendrocyte, Salmon = Endothelia.

B Unique and overlapping peptides that are upregulated (FC > 1.25, $P < 1e-2$) or downregulated (FC < .8, $P < 1e-2$) within each mouse model. Venn colors are: Red = CK-p25, Purple = 5XFAD, Yellow = Tau P301S.

C Heatmap showing phosphotyrosine peptides from the cortex of CK-p25 animals. Colors indicate fold change relative to control animals on a log2-scale. Row colors (left) indicate peptides from predicted cell-type specific proteins using the same scheme as (A).

D Correlation between phosphopeptide fold changes in the hippocampus and cortex of 2wk CK-p25 mice. Linear regression R^2 value is shown on plot.

E Cell-type enrichment analysis of phosphopeptides from CK-p25, 5XFAD, and Tau animals. Heatmap colors indicate LOE of cell-type specific proteins in the set of upregulated or downregulated phosphopeptides for each animal. Cell types are: Ast = Astrocyte; End = Endothelia; Oli = Oligodendrocyte, Mic = Microglia, Neu = Neuron.

F Phosphorylation motif logos for enriched peptides from the upregulated (FC > 1.25, $P < 1e-2$) pSer/pThr phosphoproteome of CK-p25 mice. Y-axis shows log-odds enrichment (LOE) of amino acids proximal to phosphorylation sites.

 © 2020 The Authors

Figure EV2. 5XFAD and Tau P301S phosphoproteome accessory analyses.

A Volcano plots showing changed phosphopeptides in 5XFAD animals. All tissues and time points are considered together for fold change and *P*-value calculations. Protein names are shown for changed peptides. Labels are only shown for peptides with maximum directional change from each protein. Labels are colored by predicted cell-type specific protein. Orange = Neuron, Blue = Microglia, Salmon = Endothelia.

B Quantification of transgenic amyloid precursor protein peptides identified in the hippocampus and cortex from 5XFAD and WT mice: HFEHVR and LVFFAEDVGSNK. APP / App indicates peptides mapped to both transgenic and native protein. NTF: N-terminal Fragment, $A\beta_{n\text{-}m}$: peptide mapped within Aβ.

C Heatmap showing enriched phosphotyrosine peptides in the cortex of 9 mo 5XFAD mice. Colors indicate fold change relative to control animals on a log2-scale. Row colors (left) indicate peptides from predicted cell-type specific proteins using the same scheme as (A).

D Correlation between phosphopeptide fold changes in the hippocampus and cortex of 9mo 5XFAD mice. Linear regression R^2 value is shown on plot.

E Phosphorylation motif logos for enriched peptides from the downregulated (FC < .8, P < 1e-2) pSer/pThr phosphoproteome of 5XFAD mice. Y-axis shows log-odds enrichment (LOE) of amino acids proximal to phosphorylation sites.

F Phosphopeptides associated with Δp35KI mutation in 5XFAD mice. Heatmap colors indicate log2 fold changes from hippocampus tissue. Row colors (left) indicate the number of residue positions for which peptides match the CaMKII motif: O.+Q.xO -, where 'O' indicates hydrophobic residues (FLMVI), '+' indicates positively charged residues (KR), 'x' indicates a phosphosite, and '.' indicates any residue. 'x' and '.' are uncounted for motif scores.

G Volcano plots showing changed phosphopeptides in Tau P301S mice. All tissues and time points are considered together for fold change and *P*-value calculations. Protein names are shown for changed peptides. Labels are only shown for peptides with maximum directional change from each protein. Labels are colored by predicted cell-type specific protein. Orange = Neuron, Blue = Microglia.

H Heatmap showing enriched phosphotyrosine peptides in Tau P301S 4mo hippocampus tissues. Colors indicate fold change relative to control animals on a log2-scale. Row colors (left) indicate peptides from predicted cell-type specific proteins using the same scheme as (A).

I Transgenic MAPT peptides identified in Tau P301S mice. Colored bars indicate directional changes for non-phosphorylated peptides. Red = increased, blue = decreased, grey = unchanged, light-grey = only phosphopeptides were seen in that region. Colored circles indicate phosphorylation sites that were quantified. Red circle = increased, blue circle = decreased, black circle = unchanged.

J Correlation between phosphopeptide fold changes in hippocampus tissues of 4mo and 6mo Tau P301S mice. Linear regression R^2 value is shown on plot.

© 2020 The Authors

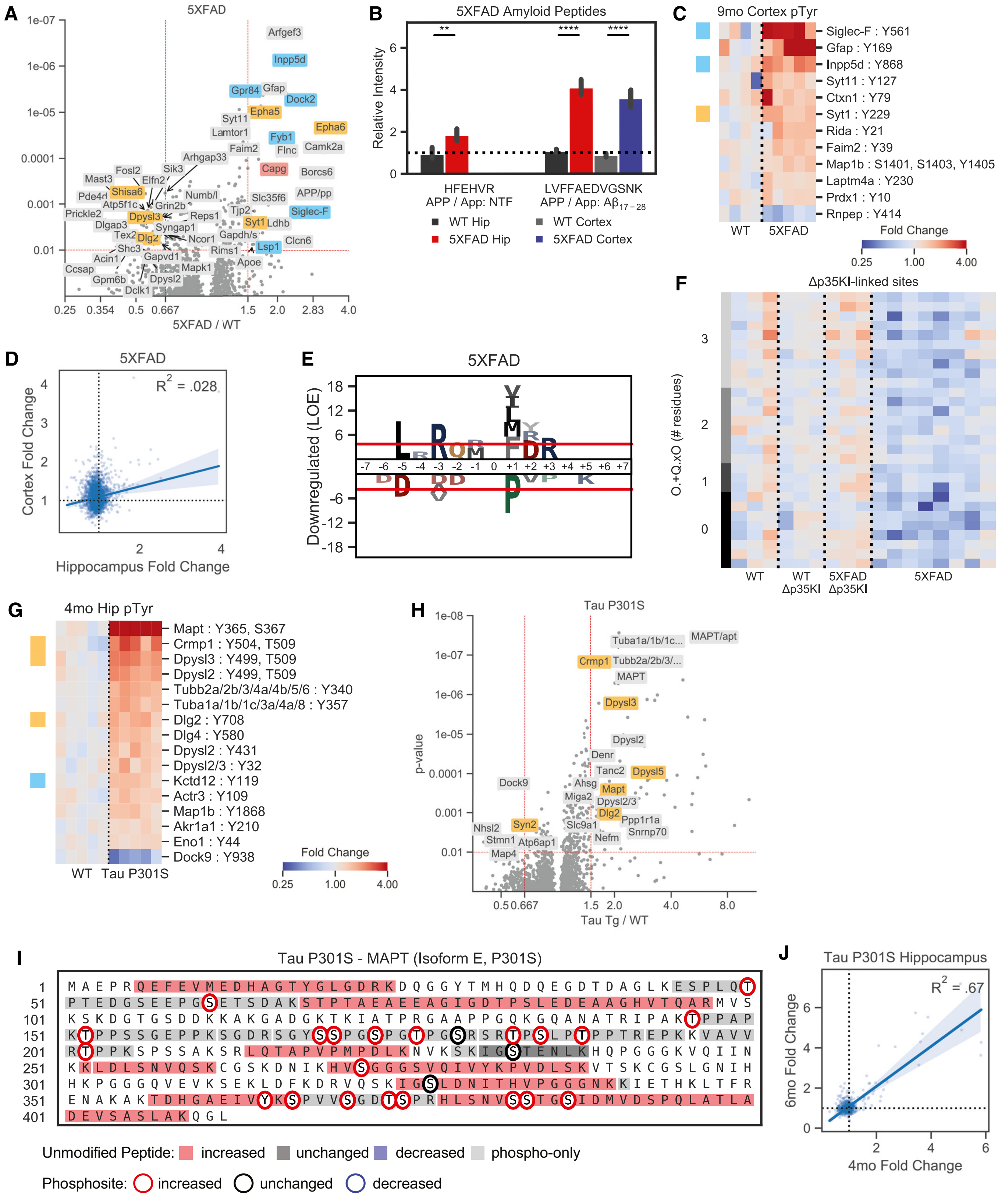
A
5XFAD
5XFAD / WT
B
5XFAD Amyloid Peptides
Relative Intensity
HFEHVR
APP / App: NTF
LVFFAEDVGSNK
WT Hip
5XFAD Hip
WT Cortex
5XFAD Cortex
C
9mo Cortex pTyr
Siglec-F : Y561
Gfap : Y169
Inpp5d : Y868
Syt11 : Y127
Ctxn1 : Y79
Syt1 : Y229
Rida : Y21
Faim2 : Y39
Map1b : S1401, S1403, Y1405
Laptm4a : Y230
Prdx1 : Y10
Rnpep : Y414
WT
5XFAD
Fold Change
D
5XFAD
Cortex Fold Change
Hippocampus Fold Change
E
5XFAD
Downregulated (LOE)
F
Δp35KI-linked sites
O.+Q.xO (# residues)
WT
WT Δp35KI
5XFAD Δp35KI
5XFAD
G
4mo Hip pTyr
Mapt : Y365, S367
Crmp1 : Y504, T509
Dpysl3 : Y499, T509
Dpysl2 : Y499, T509
Tubb2a/2b/3/4a/4b/5/6 : Y340
Tuba1a/1b/1c/3a/4a/8 : Y357
Dlg2 : Y708
Dlg4 : Y580
Dpysl2 : Y431
Dpysl2/3 : Y32
Kctd12 : Y119
Actr3 : Y109
Map1b : Y1868
Akr1a1 : Y210
Eno1 : Y44
Dock9 : Y938
WT
Tau P301S
Fold Change
H
Tau P301S
p-value
Tau Tg / WT
I
Tau P301S - MAPT (Isoform E, P301S)
Unmodified Peptide: increased unchanged decreased phospho-only
Phosphosite: increased unchanged decreased
J
Tau P301S Hippocampus
6mo Fold Change
4mo Fold Change

Figure EV2.

Figure EV3. Pathway analysis and peptide validation.

A–C Top 25 enriched gene ontologies from the phosphoproteome of (A) CK-p25 mice, (B) 5XFAD mice, and (C) Tau P301S mice.

D, E Validated peptide-spectrum matches for (D) Siglec-F pY561 (SVyTEIK) and (E) Inpp5d pY868 (LyDFVK) in CK-p25 mice. Green circles indicate predicted fragment ions. Red circles indicate abundant peaks that were not assigned a fragment ion. Orange box indicate precursor isolation window.

© 2020 The Authors

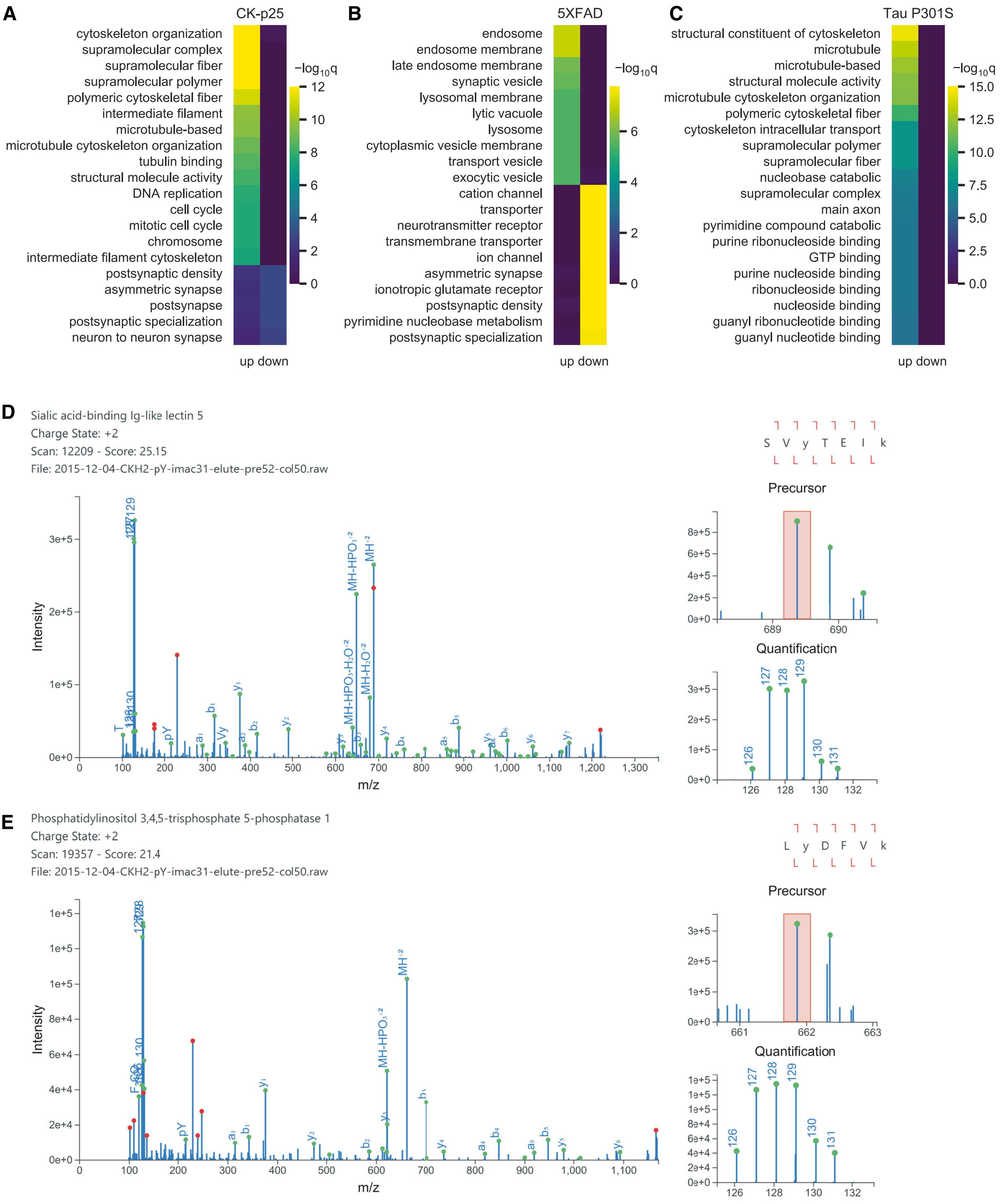

Figure EV3.

 © 2020 The Authors

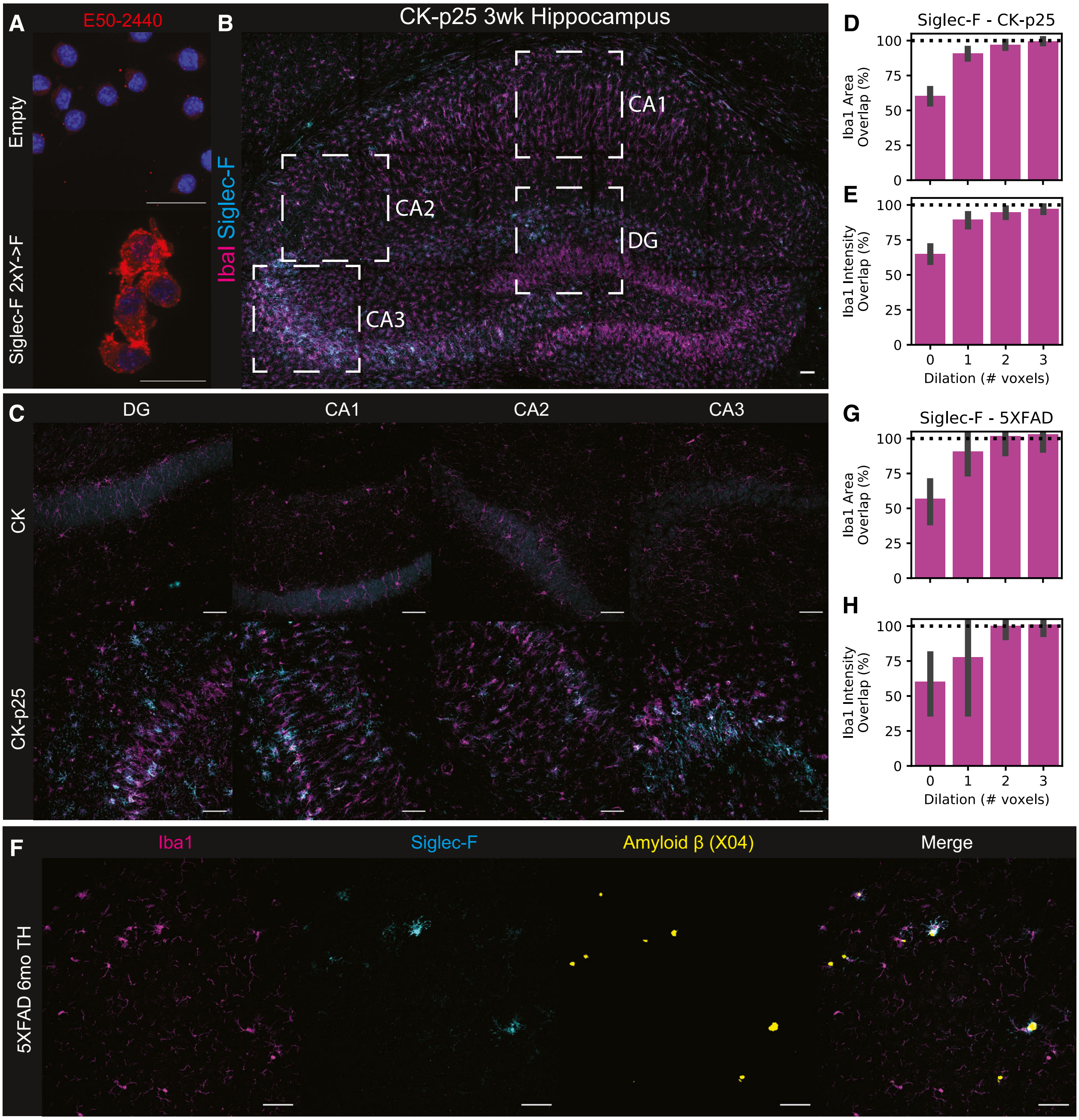

Figure EV4. Siglec-F IF antibody validation and regional scanning.

A Validation of E50-2440 antibody on BV-2 with stable Siglec-F expression. Top: Empty vector; Bottom: Siglec-F 2xY->F. Blue = 33342, Red = E50-2440. Scale bars = 20 μm.

B Immunofluorescence (IF) staining showing Siglec-F and Iba1 localization in CK-p25 3wk hippocampus tile scan view. Boxed regions indicate analyzed regions: DG, CA1, CA2, and CA3. Scale bars = 50 μm. Colors are: Magenta = Iba1, Cyan = Siglec-F. Images are max z-stack projections taken from coronal slices.

C Individual wide-field images of DG, CA1, CA2, and CA3 regions from CK (top) and CK-p25 mice (bottom).

D, E Percent of total Siglec-F mask (D) area and (E) signal intensity that overlaps with Iba1 masks that have been dilated by a variable number of pixels. ° indicates Iba1 mask was dilated prior to Boolean overlap calculation. 1 voxel ≅ 2.4 μm. Values are calculated from CA1, CA2, CA3, and DG image regions from CK-p25 animals using the same image set as Figure 1C.

F Wide-field image of Siglec-F, Iba1, and Aβ (Methoxy X04) signal across in the thalamus (TH) of 6 m.o. 5XFAD mice.

G, H Percent of total Siglec-F mask (G) area and (H) signal intensity that overlaps with Iba1 masks that have been dilated by a variable number of pixels. Legend is same as (D-E). Values are calculated from thalamic, cortical, and hippocampal regions from 5XFAD animals using the same image set as Figure 2F.

© 2020 The Authors	

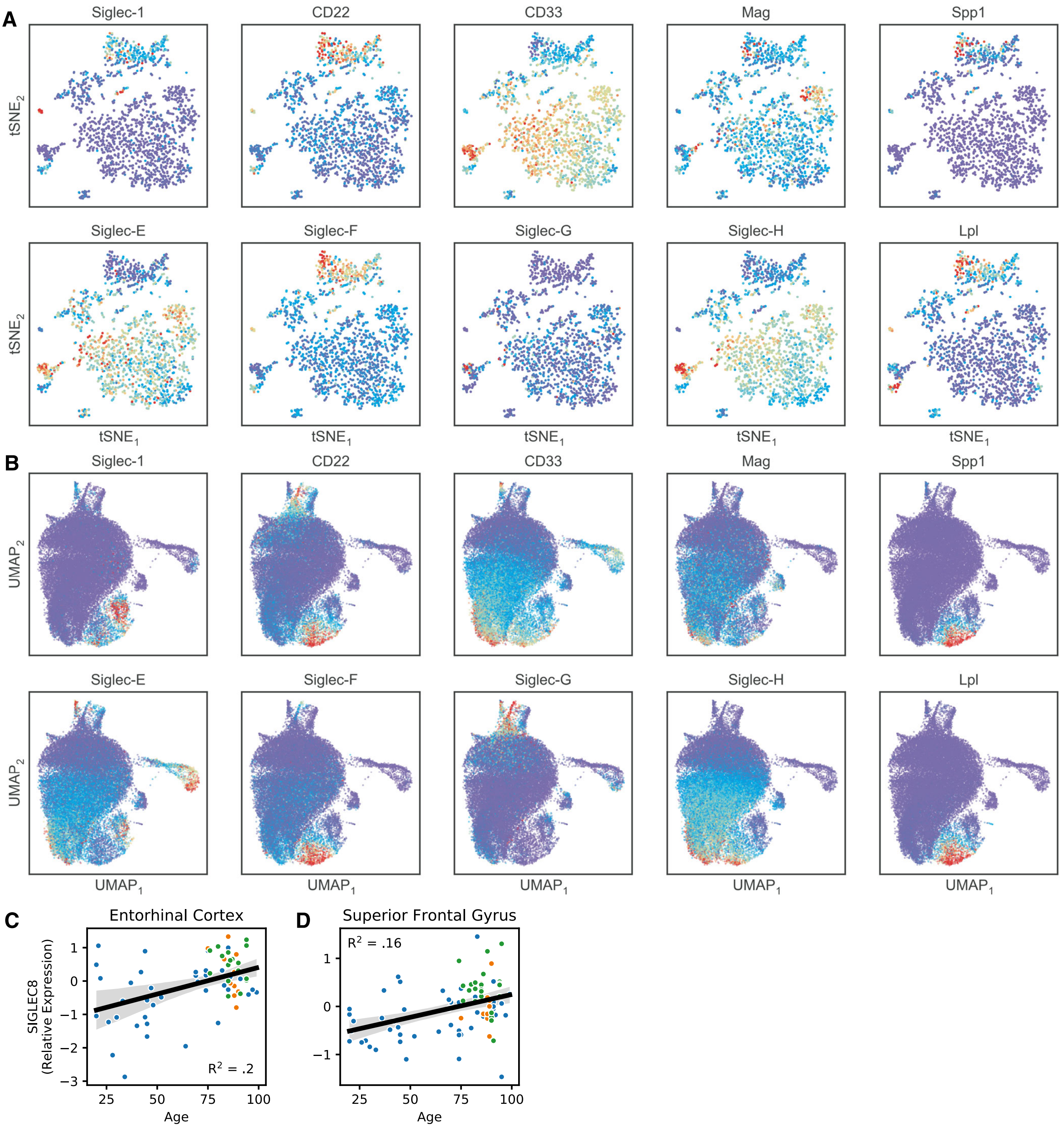

Figure EV5. Siglec expression patterns in microglial scRNA-Seq and bulk RNA-seq datasets.

A Reanalysis of Siglec expression patterns in CK-p25 microglia. Expression levels were imputing using MAGIC and plotted using the tSNE coordinates from the original publication. Late response MHC-II^{+} microglia (top cluster, marker genes: Spp1, Lpl) show increased Siglec-F expression compared with homeostatic microglia (lower right cluster).

B Reanalysis of Siglec expression patterns in 5XFAD microglia. Expression levels were projected onto two dimensions using UMAP and imputed using MAGIC. Disease-associated microglia (lower center, marker genes: Spp1, Lpl) show increased Siglec-F expression compared with homeostatic microglia (center).

C, D Relative RNA abundances for Siglec-8 in (C) entorhinal cortex and (D) superior frontal gyrus post-mortem patient tissue. Blue = ND; Yellow = MCI; Green = AD. Linear regression R^2 and P-values are shown on plots.

© 2020 The Authors

Figure EV6. Siglec-8 IF antibody validation and accessory analyses.

A IF staining for Siglec-5 (top) and Siglec-8 (bottom) in late-onset AD patient tissue (LOAD; Case ID: 03-06). Show are merge (Left; Red = Iba1, Green = Siglec, Blue = 488 background autofluorescence), background-corrected merge (Middle; Red = Iba1, Green = Siglec / 488 background), and background-corrected Siglec alone (Right). Scale bars = 50 μm.

B Antibody validation of ab198690 on BV-2 with induced Siglec-8 (48 h dox). Top: Siglec-8; Bottom: Siglec-8 2xY->F. Blue = Hoechst 33342, Red = ab198690. Scale bar = 20 μm.

C–E Representative IF images for Iba1, Siglec-8, MHC-II, and Aβ (Methoxy X04) in tissue from patients. Shown are: (C) white matter (WM) region of a LOAD patient (Case ID: 01-43); (D) grey matter (GM) region from a LOAD patient (Case ID: 03-06); and (E) GM region from an early-onset AD patient (Case ID: 00-22).

F, G Percent of total Siglec-8 mask (F) area and (G) signal intensity that overlaps with Iba1, MHC, and the union ($\bigcup$) of Iba1 and MHC. ° indicates mask were dilated in by a given number of pixels prior to Boolean overlap calculation. 1 voxel ~ 2.4 μm. Values are median stack values from $n = 3$ selected 20x wide-field images.

H Percent area coverage of Siglec-8 localized to Iba1 or MHC in grey matter (GM) and white matter (WM) across all analyzed images.

I Percent area coverage of Siglec-8 localized to Iba1 compared to total Aβ volume in each field of view across all analyzed images. Linear regression R^2 value is shown for images with ≥ 10 μm^2 total amyloid volume.

© 2020 The Authors

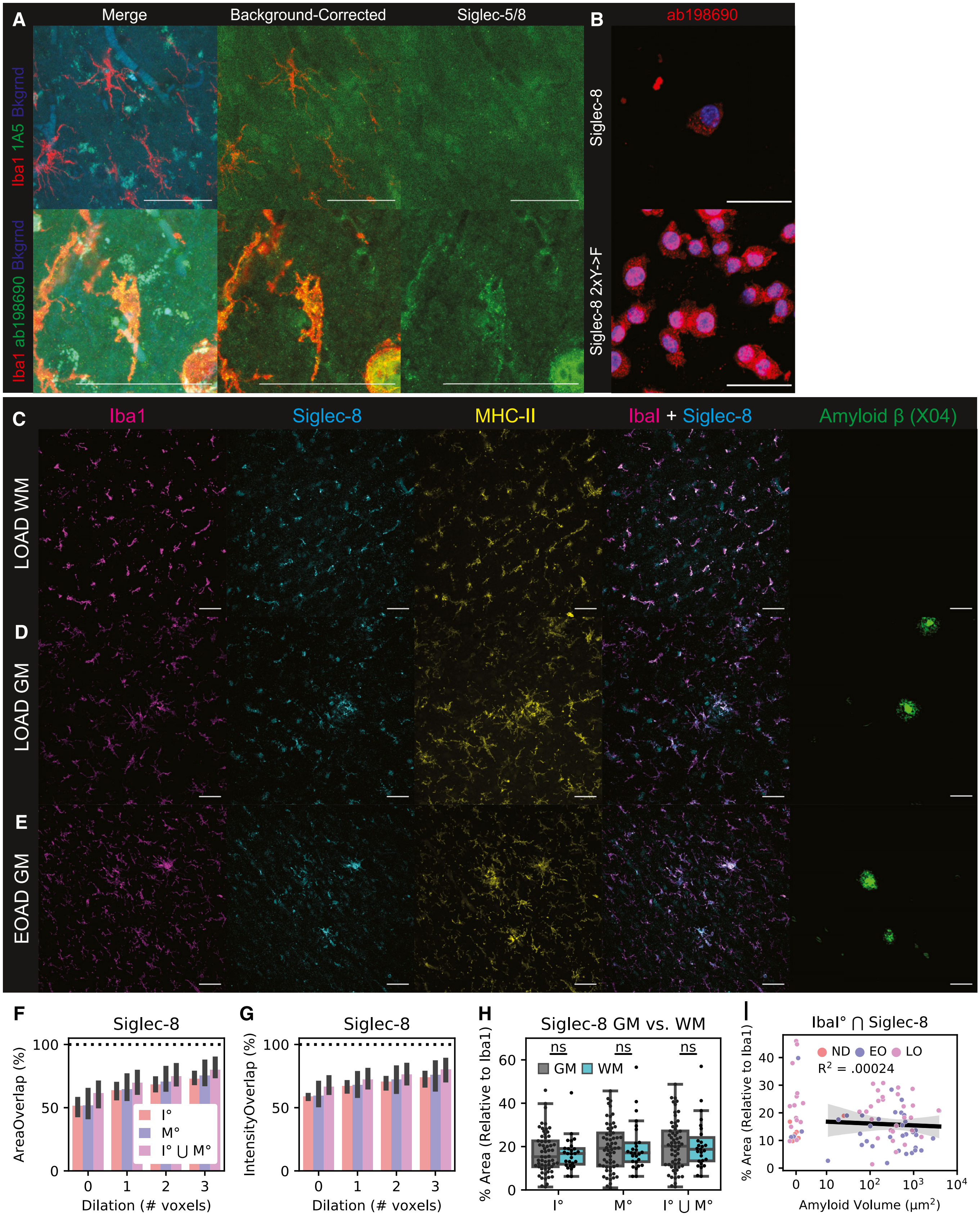

Figure EV6.

© 2020 The Authors

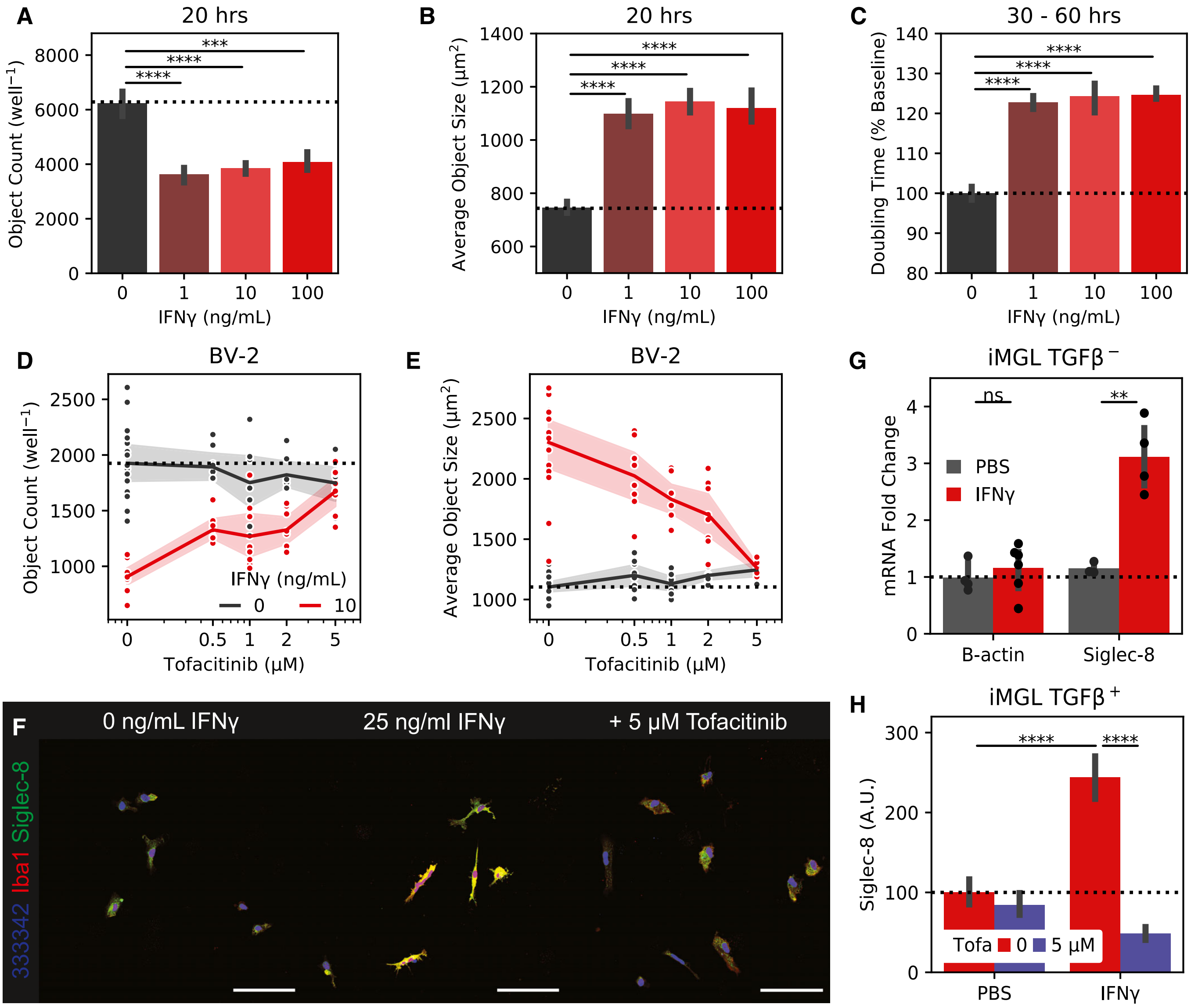

Figure EV7. BV-2 and iMGL IFNγ and tofacitinib accessory experiments.

A–C Incucyte (A) object count, (B) object size, and (C) doubling time for BV-2 cells treated with 1–100 ng/ml IFNγ. (A+B) are calculated at 20 h while (C) is calculated from confluency changes between 30 and 60 h.

D, E Incucyte (D) object count and (E) object size for BV-2 cells co-treated with 10 ng/ml IFNγ and 0–5 μM tofacitinib.

F 20x wide-field images of iMGLs treated with IFNγ or PBS and tofacitinib. Siglec-8 fluorescence is only show on non-nuclear regions. Colors are: Red = Iba1, Green = Siglec-8, Blue = Hoechst 33342. Scale bars = 100 μm.

G qPCR quantification of Siglec-8 from iMGLs treated with 25 ng/ml IFNγ or PBS.

H Quantification of Siglec-8 on iMGLs treated with IFNγ or PBS and tofacitinib. iMGLs were matured with TGFβ before IFNγ stimulation.

© 2020 The Authors

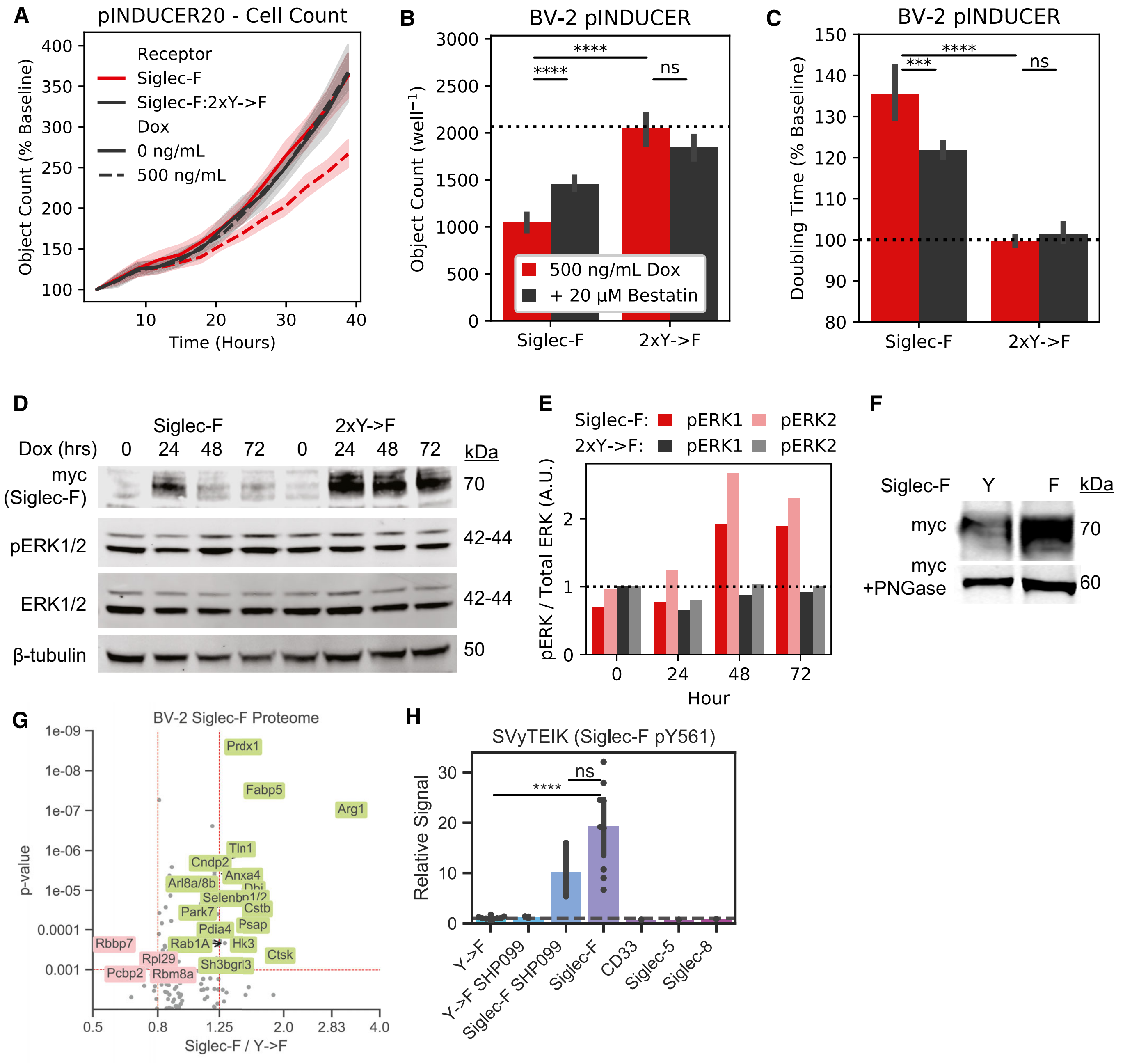

Figure EV8. BV-2 Siglec expression accessory data.

A Incucyte object count for BV-2 cells expressing inducible Siglec-F constructs. Cell counts for are normalized to the starting count in each imaging region.

B, C Incucyte quantification of (B) object count and (C) confluency doubling time for BV-2 cells expressing inducible Siglec-F and optionally co-treated with 20 μM Bestatin.

D Western blot detection of Siglec-F-myc, pERK1/2, total ERK, and β-tubulin in BV-2 cells induced to express Siglec-F for 0–72 h.

E Quantification of Erk1, Erk2, pErk1 / total Erk1, and pErk2 / total Erk2 for bands shown in (D).

F Western blot detection of myc-tagged Siglec-F +/- PNGase.

G Volcano plot showing proteomic changes induced by stable Siglec-F expression in BV-2 cells. Only proteins with ≥ 2 unique peptides that each were seen in ≥ 2 scans are shown.

H Quantification of Siglec-F pY561 (SVyTEIK) across BV-2 lines analyzed.

 © 2020 The Authors
